# Association between orthostatic blood pressure dysregulation and geriatric syndromes: a cross-sectional study

**DOI:** 10.1186/s12877-022-02844-8

**Published:** 2022-02-26

**Authors:** Frédéric Roca, Kevin Rougette, Louise Zmuda, Gabrielle Noel, Solène Larose, Mathilde Bordage, Philippe Chassagne

**Affiliations:** Department of Geriatrics, Rouen University Hospital, 76000 Rouen, France

**Keywords:** Orthostatic hypertension, Orthostatic hypotension, Dementia, Frailty, Geriatric syndromes

## Abstract

**Background:**

Orthostatic blood pressure dysregulation, including orthostatic hypotension (OH) and orthostatic hypertension (OHT), is common in the elderly. The association between OH and, to a lesser extent, OHT with geriatric syndromes is controversial and little investigated.

Our objective was to assess the association between orthostatic blood pressure dysregulation and geriatric syndromes in an ambulatory outpatient population.

**Methods:**

This observational study included all outpatients for whom a one-visit comprehensive geriatric assessment was performed during a year. OH was defined as a decrease of at least 20 mmHg in systolic blood pressure (SBP) and/or 10 mmHg in diastolic blood pressure (DBP) after 1 or 3 min of standing. OHT was defined as an increase of more than 20 mmHg in SBP after 1 or 3 min of standing. Comorbidities, drugs regimen, a history of previous falls, nutritional, frailty, functional and cognitive status were compared between patients with OHT or OH and controls (NOR).

**Results:**

Five hundred thirty patients (mean age: 82.9 ± 5.1 years) were included. 19.6% had an OH and 22.3% an OHT. OHT patients were more frequently female, had more diabetes and a lower resting SBP than patients with NOR. OH patients had a higher resting SBP than NOR. After adjusting for age, sex, resting SBP and diabetes, OHT was associated with a low walking speed (OR = 1.332[1.009–1.758]; *p* = 0.043) and severe cognitive impairment at MMSe score (OR = 1.629[1.070–1.956]; *p* = 0.016) compared to NOR. Conversely, OH was associated with a lower grip strength (*p* = 0.016) than NOR.

**Conclusion:**

OHT and OH are common in elderly but associated with different geriatric phenotypes.

**Supplementary Information:**

The online version contains supplementary material available at 10.1186/s12877-022-02844-8.

## Introduction

Orthostatic blood pressure dysregulation is a common condition in the elderly population and its frequency increases with age. The prevalence of orthostatic hypotension (OH) is about 5% in adults but rises to 30% in the community-dwelling older population aged over 65 years and reached up to 65% in a very old patients when systematic and repeated measurements are performed [1–4]. Many causes or mechanisms such as dysautonomia secondary to diabetes mellitus or Parkinson’s disease, dehydration or polymedication, especially with antihypertensive and psychotropic drugs, explain this increased prevalence of OH in the elderly [2]. OH in elderly should be considered a poor indicator of health. Thus, OH is associated with cardiovascular comorbidities, such as ischemic stroke, coronary artery disease, and peripheral vascular disease, and with cardiovascular and all-cause mortality [5–8]. An association between OH and geriatric syndromes such as falls, malnutrition and functional dependency, or common illnesses such as depression or neurocognitive disorders, have been described, although the mechanisms of these relationships are still debated [8–18]. In an Italian cohort of 510 old people, OH was significantly associated with disability, mortality and higher hospitalization rates according to the frailty status of patients [13].

Orthostatic hypertension (OHT), that seems as frequent as OH, has been less investigated. The prevalence of OHT is estimated at 2.4% in young adults but ranges from 4 to 28% in the elderly, especially in those with hypertension or diabetes [3, 8, 19, 20]. The definition of OHT is not consensual. By analogy with OH, a 20 mmHg increase in systolic blood pressure (SBP) after 1 or 3 min of standing is a widely accepted definition [21]. OHT is associated with excess cardiovascular mortality, coronary heart disease and ischemic stroke [3, 6–8, 22]. Thus, in a cohort of nursing home residents, the cardiovascular mortality in patients with OHT was similar to those with OH, near to 25% at 2 years of follow-up, but significantly higher than those without orthostatic blood pressure dysregulation [8]. Moreover, in some studies performed in “young” elderly populations with few geriatric syndromes or, conversely, in very frail nursing home residents, OHT was inconsistently associated with cognitive decline, frailty or disability [8, 15, 23, 24]. Thus, there is poor evidence that geriatric syndromes, that are poor prognosis multifactorial conditions characterizing aging people, are associated with orthostatic blood pressure dysregulation in an elderly community-dwelling population.

The aim of this study was to evaluate the association between orthostatic blood pressure dysregulation and geriatric syndromes in elderly outpatients followed in a geriatric tertiary care center.

### Study population and Methods

We prospectively included all outpatients addressed in a day-hospital unit from a geriatric department, from January 1, 2017 to December 31, 2017. In this interdisciplinary outpatient clinic, a trained staff including nurses, neuropsychologists, social workers, physiotherapists and geriatricians performed a comprehensive geriatric assessment based on recommendations in order to explore at least 8 domains [25]. Patients were mainly referred from the community by their general practitioner who was consulted if required to validate the clinical or functional characteristics collected. Patient was always accompanied by their main familial caregiver.

For each patient, the SBP, the diastolic blood pressure (DBP) and the heart rate (HR) were measured using an automatic blood pressure monitor (Carescape V100, Dinamap Technology, GE Health Care® and Mindray Datascope Duo®) with an adapted cuff in a supine position after at least 5 min of rest and after 1 and 3 min of standing. OH was defined as a decrease of at least 20 mmHg in SBP and/or 10 mmHg in DBP after 1 or 3 min while standing according to the French hypertension society criteria [26]. OHT was defined as an increase of more than 20 mmHg in SBP after 1 or 3 min in standing position [21]. Patients with both OH and OHT (at 1 and 3 min) were excluded (*n* = 6).

Demographical data (age, sex) and comorbidities such as a history of diabetes mellitus, hypertension, atrial fibrillation, heart failure, stroke, coronary artery disease, peripheral vascular disease and/or carotid atheroma were listed from medical charts. Vascular impairment was defined as a composite of history of stroke, coronary artery disease, peripheral vascular disease or documented carotid atheroma. Glomerular filtration rate (GFR) was estimated by the CKD-Epi formula and chronic kidney disease was defined as a GFR lower than 30 ml/min/m^2^.

Antihypertensive drugs regimen was detailed.

The following geriatric parameters were evaluated:

- Falls, at least one self-reported fall within the last 6 months,
- Walking speed, a walking speed over 4 m ≤ 0.8 m/s was considered as slow and as a marker of frailty [27],
- Hand-grip strength, assessed with Jamar dynamometer. A low strength was defined according to threshold depending on sex and was considered as probable sarcopenia. If associated with low walking speed, patients were considered as highly probable sarcopenia [27],
- Basic activities of daily living (BADL) and instrumental activities of daily living (IADL), 6-items and 8-items assessments respectively. A loss in one or more BADL defined disability,
- Short version of the Geriatric Depression Scale (mini-GDS), a score of one or more was associated with a high probability of depression. A history of depression was also recorded,
- Malnutrition, defined by a body mass index (BMI) lower than 21 kg/m2 and/or an albuminemia lower than 35 g/L,
- Mini-Mental State examination (MMSe) score and the clock drawing test (French GRECO version) was also performed to assess global cognitive performance [28]. MMSe was considered pathological when lower than threshold defined according to age and socio-educative level [28]. Severity of cognitive impairment was defined by the MMSe score as mild (MMSe > 20), moderate (11 < MMSe < 20), or severe (MMSe < 10). The diagnosis of dementia was based on the DSM-V criteria.
- Statistical analyses:
- The population was divided into 3 groups: OHT, OH, or the absence of changes in orthostatic blood pressure (Normal orthostatic response, NOR). Quantitative variables are presented as mean ± SD and qualitative variables as percentage. Groups were compared using a one-way ANOVA, followed by a Bonferonni test for two-by-two comparisons for the quantitative variables and by a Chi-square test for the qualitative variables. For ordinal qualitative variables whose variation is linear, a Cochran-Armitage trend test was used. Statistical significance was set at *p* < 0.05.
- Multivariate analysis of geriatric factors associated with the presence of OHT or OH was performed by logistic regression for qualitative variables and by a General Linear Model for quantitative variables after adjustment for sex, age, resting SBP and diabetes, with the NOR group as reference. The results are in Odds Ratios [95% confidence interval] and by the degree of significance p. SPSS® software (Systat; version 8; 1998) was used for all the analyses.
- In addition, we performed an exploratory analysis of the relationship between change in SBP change after 3 min of orthostatism and some geriatric factors identified in the multivariate analysis, to evaluate the impact of the magnitude of change in SBP at orthostatism on geriatric syndromes. For qualitative parameters, we described the repartition of change in SBP at orthostatism according to each category using violin plots. For quantitative parameters, we performed a second-order polynomial regression (adjusted on the same variables as the multivariate analysis) because their relationship with SBP change at orthostatism was nonlinear. These complementary analyses were performed with R Studio®, Version 1.3.1093.

## Results

### Baseline characteristics of the population

**Fig. 1:**
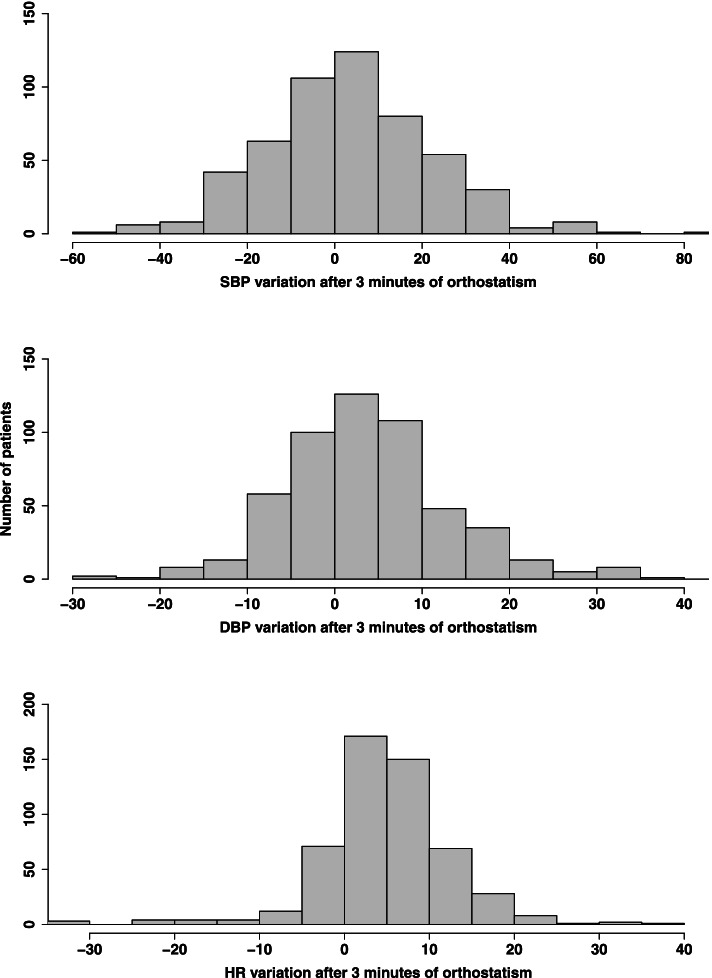
Distribution of the postural change of SBP, DBP and HR in the entire population at the third minute. *SBP* systolic blood pressure, *DBP* diastolic blood pressure, *HR* heart rate

Five hundred and thirty patients were included (351 women, 66%) with a mean age of 82.9 ± 5.1 years. In this population, 104 patients (19.6%) had OH, 118 (22.3%) had OHT and 308 (58%) had NOR. The distribution of the magnitude of postural change of SBP, DBP and HR in the entire population at the third minute is shown in Fig. 1. Hypertension and diabetes were frequent in our population (73% and 25% of patients respectively). A vascular impairment was found in 86 patients (16.2%). Mean SBP at rest was 145 ± 25.2 mmHg and mean DBP at rest was 72.7 ± 11.5 mmHg (Table 1). Four hundred and one patients had one or more anti-hypertensive drugs (75.7%), mainly angiotensin-converting enzyme inhibitors/angiotensin II receptor blockers (223 patients, 42%), beta-blockers (183 patients, 34.6%), diuretics (168 patients, 31.7%) and calcium-channel blockers (137 patients, 25.8%).

**Table 1:** Baseline characteristics of the population (*n* = 530)

Male sex	179 (33)
Age (years)	82.9 ± 5.2
**Comorbidities**	
Hypertension	387 (73)
Diabetes mellitus	132 (24.9)
Heart failure	80 (15.1)
Vascular disease	86 (16.2)
-Stroke	32 (6)
-Peripheral arterial disease	54 (10.2)
-Coronary heart disease	22 (4.2)
-Carotid stenosis	62 (11.7)
Atrial fibrillation	111 (20.9)
Chronic kidney disease	151 (28.5)
eGFR CKD-EPI (mL/min)	65.6 ± 20.3
**Resting hemodynamic parameters**	
SBP (mmHg)	145 ± 25.2
DBP (mmHg)	72.7 ± 11.5
PP (mmHg)	72.3 ± 19.9
MBP (mmHg)	96.8 ± 14.5
HR (bpm)	70.5 ± 11.8
**Antihypertensive medications**	
Treatment for hypertension	401 (75.7)
Number of antihypertensive drugs	
-1	153 (28.9)
-2	132 (24.9)
-3 or more	113 (21.3)
Type of antihypertensive medications	
-ACEi/ARB	223 (42)
-CCB	137 (25.8)
-ß-blockers	183 (34.6)
-Diuretics	168 (31.7)
-Centrally-acting antihypertensive agents	36 (6.8)
-⍺-blockers	44 (8.3)
**Geriatric characteristics**	
IADL	3.0 ± 2.6
BADL	5.0 ± 1.0
BADL ≤ 5	257 (48.6)
Depression	186 (35.5)
Mini-GDS ≥ 1	111 (40.3)
Dementia	362 (68.3)
MMSe score, /30	19.2 ± 5.8
Pathological MMS^a^	362 (72.5)
MMSe severity	
-mild	113 (31.2)
-moderate	195 (53.8)
-severe	54 (14.9)
Pathological CDT	248 (58.3)
≥ 1 self-reported fall in the last 6 months	213 (40.3)
Malnutrition^b^	97 (18)
BMI (kg/m^2^)	27.1 ± 4.8
Albumin (g/L)	39.8 ± 4.3
Sarcopenia^c^	305 (57)
Hand-grip strength (kg)	16.7 ± 7.5
Low hand-grip strength^d^	402 (75.8)
Walking speed (m/s)	0.67 ± 0.25
Low walking speed^e^	365 (69.7)

Results are expressed as mean ± SD or n (%). *eGFR CKD-EPI* estimated Glomerular Filtration Rate by CKD-EPI Formula; *SBP* Systolic Blood Pressure, *DBP* Diastolic Blood Pressure, *PP* Pulse Pressure, *MBP* Mean Blood Pressure, *HR* Heart rate, *ACEi/ARB* Angiotensin-Converting Enzyme inhibitors/Angiotensin II Receptor Blockers, *CCB* Calcium-Channel Blockers, *BMI* Body Mass Index, *BADL* Basic Activities of Daily Living, *IADL* Instrumental Activities of Daily Living, *Mini-GDS* 4-items Geriatric Depression Scale, *MMSe* Mini-Mental State Examination, *CDT* French GRECO version of the Clock Drawing Test

^a^MMSe was considered pathological when lower than threshold defined according to age and socio-educative level; ^b^Malnutrition was defined as BMI lower than 21 kg/m^2^ or albuminemia lower than 35 g/L; ^c^Sarcopenia was defined as low walking speed and low hand-grip strength; ^d^Low hand-grip strength was defined according to threshold depending on sex and BMI; ^e^Walking speed < 0.8 m/sec was considered low

Mean BADL was 5.0 ± 1.0 with a half (48.7%) of the population with a loss of one or more BADL. Mean IADL was 3.0 ± 2.6. Depression was common (186 patients, 35.5%), with a pathological GDS in 40.3% of patients. 362 patients (68.3%) had dementia and the mean MMSe was 19.2 ± 5.8. A mild impairment in MMSe was observed in 113 patients (31.2%), a moderate impairment in 195 patients (53.8%), and a severe impairment in 54 patients (14.9%). Two hundred and thirteen patients (40.3%) experienced at least one self-reported fall within past 6 months. Mean BMI was 27.1 ± 4.8 kg/m2 and mean albuminemia was 39.8 ± 4.3 g/L. Malnutrition was observed in 97 patients (18%). According to criteria, 365 patients (70%) had a low walking speed, 402 (76%) a low grip strength and 305 (57%) had simultaneously these two conditions supporting highly probable sarcopenia.

### Characteristics of the population according to change in blood pressure at orthostatic position

Resting SBP was lower in the OHT group than in the NOR group, the latter itself being lower than the OH group (137.6 ± 23.4 vs. 143.9 ± 23.8 vs. 156.8 ± 27.1 mmHg, *p* = 0.051 and *p* < 0.001 respectively). Resting DBP, PP and MBP followed the same trends and are presented in Table 2. Mean changes in SBP after 1 and 3 min of orthostatic position were respectively -1 ± 8.2 and 2.24 ± 9.1 mmHg in the NOR group, -21.9 ± 12.1 and -18.7 ± 13 mmHg in the OH group and 20 ± 17.9 and 29.4 ± 12.7 mmHg in the OHT group (Table 2). There were no significant changes in HR between groups (Table 2).

**Table 2:** Hemodynamic parameters at rest and at orthostatic position

	Overall population *n* = 530	NOR *n* = 308	OH *n* = 104	OHT *n* = 118	p overall	p NOR vs OHT	p NOR vs OH	p OH vs OHT
Resting SBP, mmHg	145 ± 25.2	143.9 ± 23.8	156.8 ± 27.1	137.6 ± 23.4	** < 0.001**	0.051	** < 0.001**	** < 0.001**
Resting DBP, mmHg	72.7 ± 11.5	72.36 ± 10.9	76.4 ± 12.9	70.6 ± 11.1	**0.001**	0.516	**0.006**	**0.001**
Resting PP, mmHg	72.3 ± 19.9	71.6 ± 18.3	80.4 ± 22.8	66.9 ± 19	** < 0.001**	0.085	** < 0.001**	** < 0.001**
Resting MBP, mmHg	96.8 ± 14.5	96.2 ± 13.9	103 ± 155	93 ± 13.6	** < 0.001**	0.101	** < 0.001**	** < 0.001**
Δ syst 1', mmHg	-0.42 ± 17.9	-1 ± 8.2	-21.9 ± 12.1	20 ± 17.9	-	-	-	-
Δ syst 3', mmHg	4.19 ± 19.1	2.24 ± 9.1	-18.7 ± 13	29.4 ± 12.7	-	-	-	-
Δ dia 1', mmHg	2.87 ± 10.1	2.95 ± 6.6	-5.3 ± 10.7	9.8 ± 11.7	-	-	-	-
Δ dia 3', mmHg	4.62 ± 10.3	4.56 ± 6.8	-4.14 ± 8.8	12.5 ± 12.4	-	-	-	-
Resting HR, bpm	70.5 ± 11.8	69.84 ± 11.9	69.8 ± 10.9	72.7 ± 12	0.064	0.073	1	0.199

Results are mean ± SD. *NOR* Normal orthostatic response; *OH* Orthostatic hypotension; *OHT* Orthostatic hypertension; *SBP* Systolic Blood Pressure; *DBP* Diastolic Blood Pressure; *PP* Pulse Pressure; *MBP* Mean Blood Pressure; *Δ syst 1'* SBP variation after 1 min of orthostatism; *Δ syst 3’* SBP variation after 3 min of orthostatism, *Δ dia 1’* DBP variation after 1 min of orthostatism; *Δ dia 3’* DBP variation after 3 min of orthostatism; *HR* Heart Rate

Patients with OHT were significantly older than OH (83.9 ± 4.7 vs. 81.8 ± 5.3 years, *p* = 0.009). Patients with OHT had more diabetes than patients with NOR (32.2% vs. 21.7%, *p* = 0.025) but less vascular diseases (10.2%) than patients with OH (18.2%) and those with NOR (17.8%) (*p* = 0.005 and *p* = 0.042 respectively). The other comorbidities and patients’ characteristics were similar in the three groups (Table 3).

**Table 3:** Patients’ characteristics, comorbidities and antihypertensives medications according to change in blood pressure to orthostatic position

	NOR *n* = 308	OH *n* = 104	OHT *n* = 118	p overall	p NOR vs OHT	p NOR vs OH	p OH vs OHT
Male sex	111 (36)	43 (41.3)	25 (21.2)	0.003	0.003	0.333	0.001
Age (year)	83.0 ± 5.1	81.8 ± 5.3	83.9 ± 4.7	0.012	0.312	0.14	0.009
Hypertension	218 (70.7)	79 (76)	90 (76.3)	0.392			
Diabetes mellitus	67 (21.7)	27 (26)	38 (32.2)	0.08	0.025	0.377	0.308
Heart failure	53 (17.2)	15 (14.4)	12 (10.2)	0.188			
Vascular disease	55 (17.8)	19 (18.2)	12 (10.2)	0.018	0.042	0.163	0.005
-Stroke	19 (6.2)	7 (6.7)	6 (5.1)	0.128			
-Peripheral arterial disease	35 (11.4)	13 (12.5)	6 (5.1)	0.867			
-Coronary heart disease	10 (3.2)	8 (7.7)	4 (3.4)	0.109	**0.049**	0.755	**0.049**
-Carotid stenosis	34 (11)	12 (11.5)	16 (13.6)	0.13			
Atrial fibrillation	63 (20.4)	23 (22.1)	25 (21.2)	0.935			
Chronic kidney disease	90 (29.2)	38 (36.5)	23 (19.5)	0.768			
eGFR CKD-EPI (mL/min)	66.5 ± 19.6	62.7 ± 19.8	65.9 ± 22.3	0.26			
Treatment for hypertension	236 (76.6)	75 (72.1)	90 (76.3)	0.641			
Antihypertensive drugs per patient				0.443			
-1	84 (27.3)	29 (27.9)	40 (33.9)				
-2	84 (27.3)	19 (18.3)	29 (24.6)				
-3 or more	65 (21.1)	26 (25)	22 (18.6)				
Type of antihypertensive medications							
-ACEi/ARB	131 (42.5)	39 (37.5)	53 (44.9)	0.519			
-CCB	80 (25.9)	30 (28.8)	27 (22.9)	0.597			
-ß-blockers	109 (35.5)	37 (35.6)	37 (31.4)	0.703			
-Diuretics	105 (34.1)	29 (27.9)	34 (28.8)	0.547			
-Centrally-acting antihypertensive agents	17 (5.5)	5 (4.8)	14 (11.9)	**0.044**	**0.024**	0.78	0.061
-⍺-blockers	27 (8.7)	10 (9.6)	7 (5.9)	0.551			

Results are expressed as mean ± SD or n (%). *NOR* Normal orthostatic response, *OH* Orthostatic hypotension, *OHT* Orthostatic hypertension, *eGFR CKD-EPI* estimated Glomerular Filtration Rate by CKD-EPI Formula, *ACEi/ARB* Angiotensin-Converting Enzyme inhibitors/Angiotensin II Receptor Blockers, *CCB* Calcium-Channel Blockers;

Antihypertensive medications were similar in the three groups, except for the central antihypertensive treatments which were more frequent in the OHT group (11.9%) than in the NOR (5.5%) and the OH (4%) groups (*p* = 0.024 and *p* = 0.06 respectively) (Table 3).

A low walking speed was more frequent in the OHT group compared to the NOR and OH groups (81% vs. 67% vs. 64%; *p* = 0.005 and *p* = 0.006 respectively). Similarly, the walking speed was lower in the OHT group compared to the NOR group (0.61 ± 0.24 m/s vs. 0.68 ± 0.24 m/s; *p* = 0.02). The grip strength was significantly lower in the OHT group compared to the NOR group (14.7 ± 6.4 kg/m^2^ vs. 17.5 ± 7.7 kg/m^2^; *p* = 0.002) (Table 4).

**Table 4:** Geriatric characteristics according to change in blood pressure to orthostatic position

	NOR	OH	OHT	p overall	p NOR vs OHT	p NOR vs OH	p OH vs OHT
IADL	2.9 ± 2.6	3.3 ± 2.8	3.0 ± 2.6	0.472			
BADL	5.0 ± 1	5.0 ± 1.1	5.1 ± 0.8	0.841			
BADL ≤ 5	152 (49.3)	44 (42.7)	61 (51.7)	0.378			
Depression	104 (34)	41 (40.6)	41(35.3)	0.484			
Mini-GDS ≥ 1	66 (40.5)	18 (35.3)	27(44.3)	0.628			
Dementia	217 (70.5)	65 (62.5)	80 (67.8)	0.318			
MMSe score, /30	19 ± 5.7	19.9 ± 6	18.7 ± 6	0.269			
Pathological MMSe^a^	217 (73.6)	65 (67)	80 (74.8)	0.38			
MMSe severity				0.184	0.058	0.984	0.162
- mild	69 (31.8)	24 (37)	20 (25)				
- moderate	122 (56)	30 (46)	43 (53.8)				
- severe	26 (12)	11 (17)	17 (21.3)				
Pathological CDT	141 (58.3)	48 (53)	59 (63.4)	0.382			
≥ 1 self-reported fall in the last 6 months	120 (39)	50 (48)	43 (36.7)	0.182			
Malnutrition^b^	55 (17.8)	20 (19.2)	22 (18.6)	0.946			
BMI (kg/m^2^)	27 ± 4.6	27 ± 4.7	27.8 ± 5.2	0.165			
Albumin (g/L)	40 ± 4.4	39.3 ± 4	39.5 ± 4.5	0.255			
Sarcopenia^c^	169 (54.8)	60 (57.7)	76 (64.4)	0.204			
Hand-grip strength (kg)	17.5 ± 7.7	16.3 ± 7.9	14.7 ± 6.4	**0.003**	**0.002**	0.502	0.36
Low hand-grip strength^d^	225 (73)	85 (81.7)	92 (78)	0.298			
Walking speed (m/s)	0.68 ± 0.24	0.67 ± 0.26	0.61 ± 0.24	**0.024**	**0.02**	1	0.165
Low walking speed^e^	205 (67)	66 (65)	94 (81)	**0.009**	**0.005**	0.672	**0.006**

Results are expressed as mean ± SD or n (%). *NOR* Normal orthostatic response, *OH* Orthostatic hypotension, *OHT* Orthostatic hypertension, *BADL* Basic Activities of Daily Living, *IADL* Instrumental Activities of Daily Living, *Mini-GDS* 4-items Geriatric Depression Scale, *MMSe* Mini-Mental State Examination, *CDT* French GRECO version of the Clock Drawing Test. ^a^MMSe was considered pathological when lower than threshold defined according to age and socio-educative level, ^b^Malnutrition was defined as BMI lower than 21 kg/m^2^ or albuminemia lower than 35 g/L, ^c^Sarcopenia was defined as low walking speed and low hand-grip strength, ^d^Low hand-grip strength was defined according to threshold depending on sex and BMI, ^e^Walking speed < 0.8 m/sec was considered low

There was no significant difference between the 3 groups (and according to the repartition of the change in SBP at orthostatism) concerning the other geriatric syndromes, such as disability in BADL, malnutrition, depression, sarcopenia or falls. Consistently, the distribution of the population according to change in SBP at orthostatism was similar regardless disability in BADL, malnutrition or depression (Supplementary Figure). Neither the proportion of patients with a history of dementia nor the MMSe measurement (19 ± 5.7 vs. 19.9 ± 6 vs. 18.7 ± 6, *p* = 0.269) were different between the three groups. The prevalence of a severe impairment of the MMSe tended to be higher in the OHT group compared to NOR (21.3% vs. 12%) (Table 4).

### Multivariate analysis

**Fig. 2:**
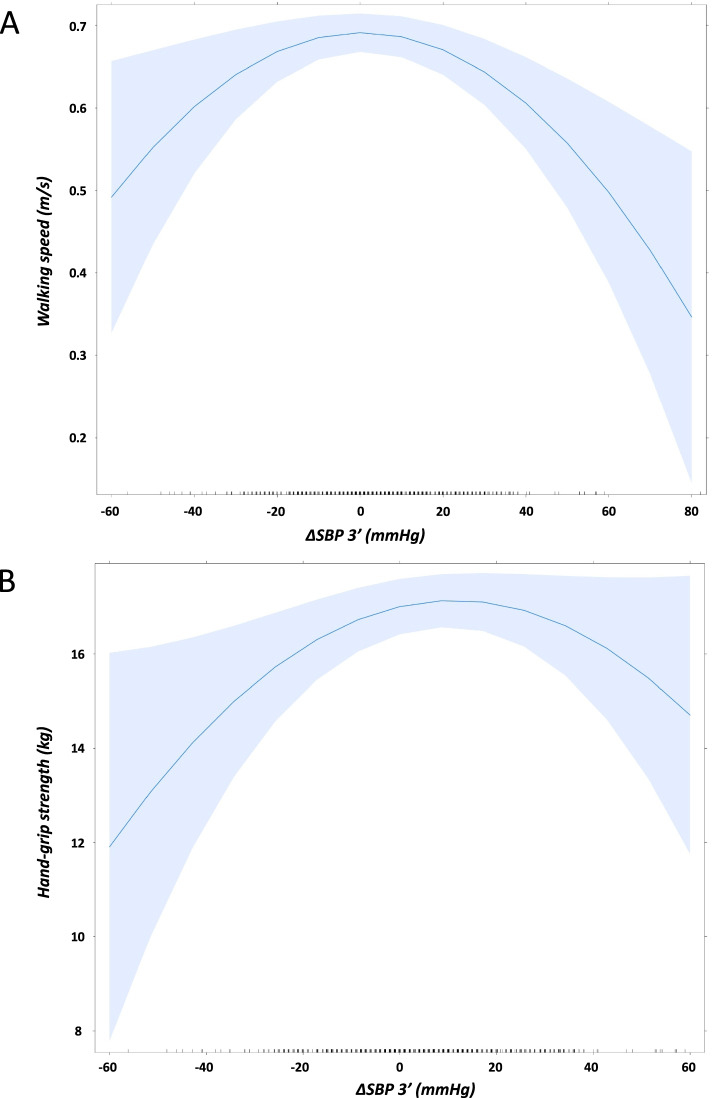
Inverse J-Shaped relationship between change in SBP at orthostatism and walking speed (**A**) or hand-grip strength (**B**). There was an inverse second-order polynomial relationship between change in SBP after 3 min of orthostatism and walking speed (ßadjusted = -0.71 [-1.2; -0.25]; *p* = 0.003) and hand-grip strength (ßadjusted = -12 [-23; -1.1]; *p* = 0.032). The 95% confidence interval of each relationship is shown by the shaded area. *∆SBP 3’* change in systolic blood pressure after 3 min of orthostatism

**Fig. 3:**
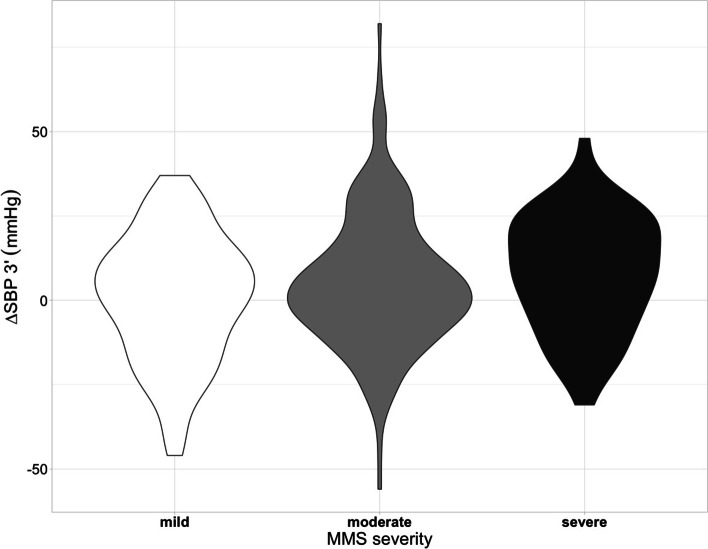
Distribution of the magnitude of change in SBP after 3 min of orthostatism according to the MMSe severity. This violin-plot shows that the highest population density of patients with severe MMSE is centered on an orthostatic SBP change value of + 20 mmHg, while that of patients with mild or moderate impairment is close to 0 mmHg. *∆SBP 3’* change in systolic blood pressure after 3 min of orthostatism

After adjustment for cofounding factors sex, age, resting SBP and diabetes, OHT was associated with a low walking speed (OR = 1.332[1.009–1.758]; *p* = 0.043) and with a severe impairment at MMSe score (OR = 1.629[1.070–1.956]; *p* = 0.016). Consistently, we observed a significant inverse J-shaped relationship between walking speed and change in SBP after 3 min of orthostatism (ßadjusted = -0.71 [-1.2; -0.25]; *p* = 0.003), more pronounced for positive than negative changes. Moreover, the highest population density of patients with severe MMSE was centered on an orthostatic SBP change value of + 20 mmHg, while that of patients with mild or moderate impairment was close to 0 mmHg (Figs. 2A and 3).

Conversely to the univariate analysis, OH was associated with a low grip strength (OR = 1.447[1.070–1.956]; *p* = 0.016) (Table 5). Consistently, we observed a significant inverse J-shaped relationship between hand grip strength and change in SBP after 3 min of orthostatism (ßadjusted = -12 [-23; -1.1]; *p* = 0.032), more pronounced for negative than positive changes (Fig. 2B).

**Table 5:** Geriatric characteristics associated with OH and OHT (multivariate analysis)

	NOR	OHT	p	OH	p
Vascular disease^a^	1 [ref]	0.743 [0.563—0.979]	**0.035**	1.157 [0.9—1.487]	0.254
Low walking speed^b^	1 [ref]	1.332 [1.009—1.758]	**0.043**	1.002 [0.778—1.292]	0.985
Hand-grip strength, /1 kg	1 [ref]	1.051 [0.799—1.382]	0.722	1.447 [1.070—1.956]	**0.016**
Moderate impairment in MMSe*	1 [ref]	0.827 [0.578—1.183]	0.298	0.773 [0.520—1.149]	0.202
Severe impairment in MMSe*	1 [ref]	1.629 [1.024—2.591]	**0.039**	1.317 [0.781—2.221]	0.301

*NOR* Normal orthostatic response, *OHT* Orthostatic hypertension, *OH* Orthostatic hypotension, *MMSe* Mini-Mental State examination. ^a^Vascular disease was a composite of stroke, peripheral arterial disease, coronary heart disease or carotid stenosis, ^b^Walking speed lower than 0.8 m/s was considered low

Multivariate analysis was performed after adjustment for age, sex, resting systolic blood pressure and diabetes by using logistic regression for qualitative variables and general linear model for quantitative variables with the NOR group as reference. Results are Odds Ratio [IC 95%]. *Reference group was mild impairment in MMSe (≥ 20*)*

## Discussion

In an ambulatory population setting of 530 old people referred for a comprehensive geriatric assessment, 42% had an orthostatic blood pressure dysregulation, either OH or OHT. A low grip strength was more frequently observed in people with OH. In people with OHT, a low walking speed and a severe impairment at MMSe score were more frequent.

Contrary to previous studies that focused on younger ambulatory population or institutionalized patients, we included an ambulatory population over 75 years old [8, 15, 23]. Moreover, most of these studies focused on mortality and cardiovascular consequences of orthostatic blood pressure changes, mainly OH [3, 6, 7, 22, 29–35], but less on the geriatric syndromes [4, 36–39].

We performed a comprehensive geriatric assessment and used recommended criteria to define OH and the most accepted definition of OHT [21, 26]. According to this method, we confirmed the high prevalence of OH but also of OHT, which has been less investigated. Thus, in middle-aged community-dwelling population, OHT is rare (2.4%) but reaches 9 to 28% in elderly ambulatory or institutionalized populations [3, 8, 21]. One study found very low prevalence of OHT in elderly patients, but orthostatic position was obtained using the sit to stand method rather than supine to standing, potentially risking an underestimate in OHT [20].

In our work, high resting blood pressure was associated with OH whereas low resting blood pressure with OHT. Some authors suggest that this last condition could be considered as masked hypertension [21]. Arterial stiffness, dysregulation of the autonomic nervous system, or endothelial dysfunction are suspected mechanisms that could explain the link between hypertension and OHT or OH [2, 21, 40]. Thus, as done in our work, studies about changes in orthostatic blood pressure must consider a history of hypertension or blood pressure values at rest when analyzing the effects of OH and OHT.

Our study identified that some frailty indicators such as low walking speed or low hand grip strength were associated with orthostatic blood pressure dysregulation. In addition, our exploratory analysis showed an inverse J-shaped relationship between these parameters and change in SBP at orthostatism, suggesting that the greater the decrease or increase in SBP, the greater the impairment. Walking speed alone has been proposed to quickly and easily assess frailty, with a cut-off of 0.8 m/s [41]. From our results, OHT was associated with a low walking speed, even after adjusting for confounding factors such as hypertension, diabetes, gender or age. To our knowledge, few studies found an association between impaired physical performance and OHT. Toba et al. found an association between OHT and the Kihon Checklist frailty criterion that include both subjective (fatigue, daily living status, mental status) and objective criteria (muscle mass, walking speed on 4 m, timed up and go test, hand-grip strength and monopodal support) [24]. However, this association was observed for the subjective criteria and not for the objective criteria of physical performance. An association between frailty and hemodynamic variations in orthostatic position or dysautonomia has also been described, suggesting that these mechanisms could explain the relationship between frailty and OHT [42, 43]. In fact, sympathetic activation, one of the main mechanisms of OHT has been observed in frail patients [43].

The link between orthostatic blood pressure dysregulation and hand-grip strength is more complex to analyse. Age and gender are major determinants of the hand-grip strength explaining why the association between low muscle strength and OHT disappeared after adjustment for confounding factors. Conversely, this adjustment shows the link between low grip strength and OH. This result is probably insufficient to conclude definitively on a relationship between frailty or sarcopenia and OH, especially since frailty does not seem to be associated with OH in literature but their deleterious effects could act synergistically on the patients’ prognosis [13, 44]. In accordance, Romero-Ortuno et al. found that despite a similar drop in SBP between non frail and frail patients, SBP recoverability was impaired during frailty, supporting the link between orthostatic hemodynamic disorders and frailty [42]. In addition, whether a decrease in muscle strength is a cause or a consequence of OH is unclear. The latter may itself be responsible for a loss of functional autonomy, immobility, and muscle loss, each of which being known to impair the muscle pump and thus the mechanism thwarting OH [10, 17]. On the contrary, inflammation or oxidative stress driving sarcopenia could also have a deleterious impact on cardiovascular system and thus lead to OH [45].

We did not find any association between MMSe and OH. Results in the literature are controversial but a recent meta-analysis shown that patients with OH have lower MMSe, especially in nursing home residents [16]. Thus, authors concluded that most of the studies which do not find an association between MMSe and OH are retrospective or cross-sectional studies, like ours, while studies finding an association are rather longitudinal [16]. The link between OHT and cognitive functions has been less investigated. To our knowledge, this is the first study demonstrating this association between OHT and severe cognitive impairment (MMSe < 10), even after adjusting for confounding factors. Previous works identify association between OHT and impaired executive or global cognitive functions but in younger population or in patients without severe cognitive decline [14, 23, 46]. Conversely, Agnoletti et al. and Curreri et al. [8, 15] did not find a lower MMSe score in their OHT group compared to their normal BP group, but their populations were different from ours, with a higher baseline MMSe than in our work. However, Curreri et al. found a faster cognitive decline in the OHT group suggesting that these patients could quickly reach severe impairment of MMSe. The role of HTO in cognitive decline could be explained by an increased risk of silent cerebrovascular infarction, but also by increased cerebral pulsatility or disruption of the blood–brain barrier that increase oxidative stress and endothelial dysfunction, leading to impaired cerebral blood flow autoregulation, beta-amyloid protein accumulation and neuronal damage [30, 47, 48].

### Limitations

Our work has some limits. First, its cross-sectional design does not permit to explore the temporal relationship between change in blood pressure with orthostatic position and geriatric syndromes and thus whether these syndromes are the cause or the consequence of OH or OHT. In addition, although we performed a comprehensive assessment, the design of this study does not allow us to be certain that we identified all the confounding factors. Secondary, some authors suggest that head-up tilt test could improve the diagnosis of OHT, but this method is less physiological and more difficult to systematically applied in clinical practice. Third, we did not explore mechanisms implied but it was not the focus of this study. Finally, consensual definition of OHT needs to be validated in future randomized and larger studies.

## Conclusion

Orthostatic blood pressure dysregulation is highly prevalent in an outpatient geriatric population. The geriatric profile of OH and OHT patients is different the last being more associated with severe cognitive impairment and frailty, assessed by low walking speed. This suggests that OH and OHT have different determinants and consequences. The effect of orthostatic blood pressure dysregulation on the course of geriatric syndromes needs to be investigated.

## Supplementary Information

**Additional file 1: Figure S1. **Distribution of the magnitude of change in SBP after 3 minutes of orthostatism according to the disability in BADL (A), malnutrition (B) or depression (C). These violin-plots show that the highest population density of patients with or without disability in BADL (A), malnutrition (B) or depression (C) is centered on an orthostatic SBP change value close to 0 mmHg. ∆SBP 3’: change in systolic blood pressure after 3 minutes of orthostatism, BADL: basic activities daily living.

## Data Availability

The datasets used and/or analyzed during the current study are available from the corresponding author on reasonable request.

## Abbreviations

BADL: Basic activities of daily living
BMI: body mass index
CDT: French GRECO version of the Clock Drawing Test
DBP: Diastolic blood pressure
IADL: 8-Items instrumental activities of daily living
Mini-GDS: Short version of the Geriatric Depression Scale
MMSe: Mini-Mental State examination
GFR: Glomerular filtration rate
HR: Heart rate
NOR: Normal orthostatic response
OH: Orthostatic hypotension
OHT: Orthostatic hypertension
SBP: Systolic blood pressure
