# Supplementary figures and images for: Association between orthostatic blood pressure dysregulation and geriatric syndromes: a cross-sectional study
Source: BMC Geriatr. 2022 Feb 26;22:157. doi: 10.1186/s12877-022-02844-8 (PMC8881862; doi:10.1186/s12877-022-02844-8)

A

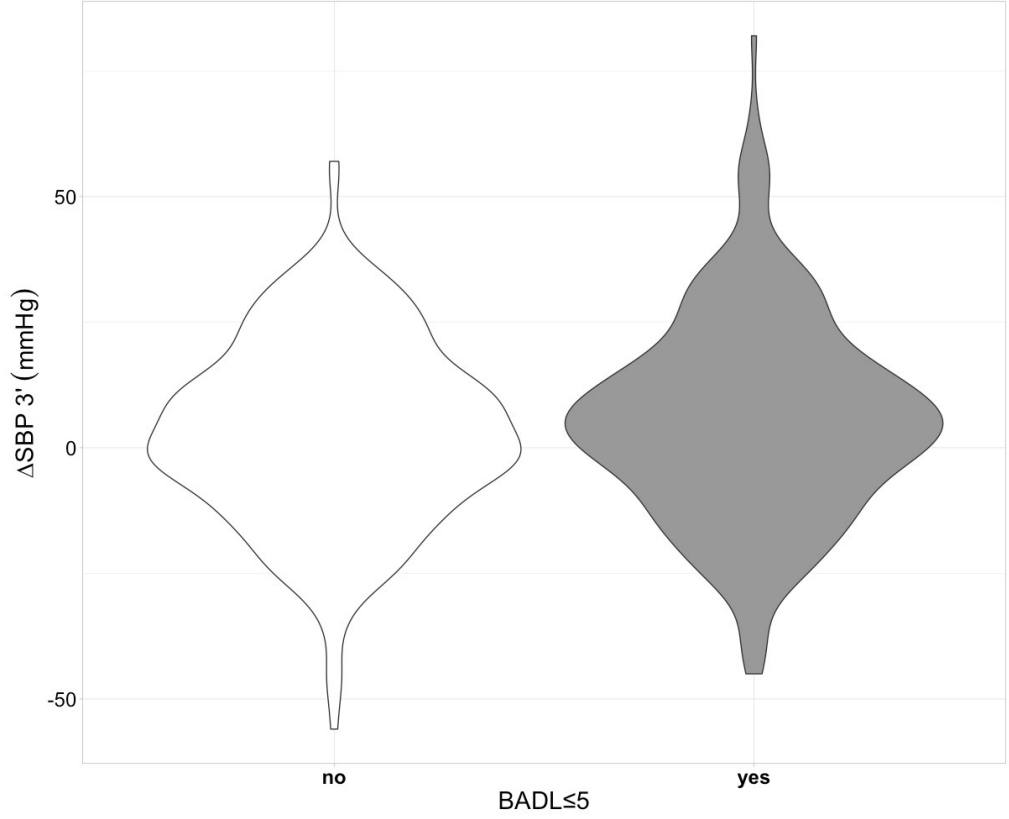

B

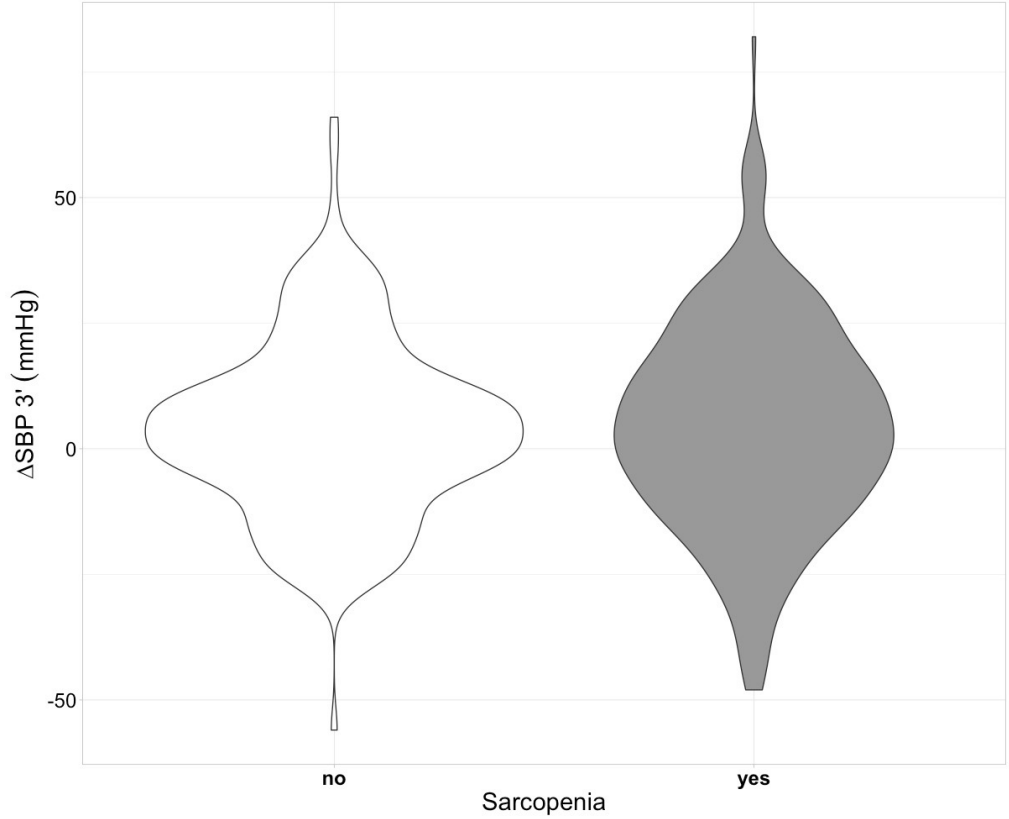

C

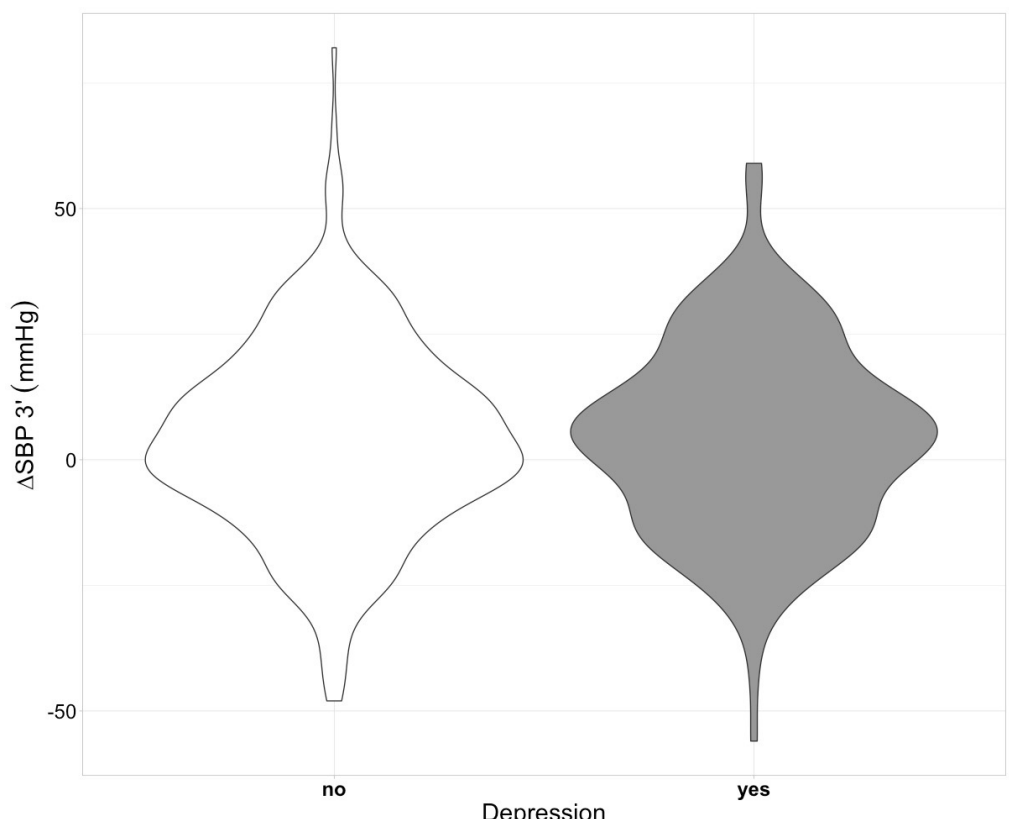

Supplement: Supplementary file 1 — Additional file 1: Figure S1. Distribution of the magnitude of change in SBP after 3 minutes of orthostatism according to the disability in BADL (A), malnutrition (B) or depression (C). These violin-plots show that the highest population density of patients with or without disability in BADL (A), malnutrition (B) or depression (C) is centered on an orthostatic SBP change value close to 0 mmHg. ∆SBP 3’: change in systolic blood pressure after 3 minutes of orthostatism, BADL: basic activities daily living. [file 12877_2022_2844_MOESM1_ESM.pdf]
